# Changes in the Gut Metabolic Profile of Gestational Diabetes Mellitus Rats Following Probiotic Supplementation

**DOI:** 10.3389/fmicb.2022.779314

**Published:** 2022-04-08

**Authors:** Qing-Xiang Zheng, Hai-Wei Wang, Xiu-Min Jiang, Li Ge, Yu-Ting Lai, Xin-Yong Jiang, Ping-Ping Huang, Fan Chen, Xiao-Qian Chen

**Affiliations:** ^1^Fujian Maternity and Child Health Hospital, Affiliated to Fujian Medical University, Fuzhou, China; ^2^Fujian Obstetrics and Gynecology Hospital, Affiliated to Fujian Medical University, Fuzhou, China; ^3^School of Nursing, Fujian University of Traditional Chinese Medicine, Fuzhou, China

**Keywords:** gut metabolites, gestational diabetes mellitus rats, probiotic supplements, glucose and insulin resistance, host metabolism

## Abstract

The roles of gut microbiota and metabolomics in women with gestational diabetes mellitus (GDM) are not well understood. This study investigated the gut metabolomic profiling of GDM rats and GDM rats treated with probiotic supplements. Associations between gut metabolites and microbiota were also studied in GDM rats. Liquid chromatography–mass spectrometry was used to detect gut metabolites in GDM rats and GDM rats treated with probiotic supplements of 0.5 g (low-dose group) or 1 g (high-dose group) for 15 days. Each gram of probiotic supplement contained 5 × 10^7^ colony-forming units (CFU) of *Lactobacillus rhamnosus LGG* and 1 × 10^8^ CFU of *Bifidobacterium animalis subspecies lactis* Bb12. The association between gut metabolites and microbiota in GDM rats was investigated using Spearman’s correlation. Finally, 10 rats in the normal pregnant group, eight rats in the GDM model group, eight GDM rats in the low-dose probiotics group, and nine GDM rats in the high-dose probiotics group were further studied. Serum parameters and pancreatic and colon histology were significantly changed in GDM rats, and these were restored using probiotic supplements. In total, 999 gut metabolites were detected in the feces, and GDM rats were distinguished from normal rats. The levels of 44 metabolites were increased in GDM rats, and they were alleviated using probiotic supplements. Changes in metabolites in GDM rats were associated with amino acids and bile acids metabolism signaling pathways. Furthermore, changes in metabolites after probiotic supplementation were associated with porphyrin and chlorophyll metabolism pathways. We found that the *Allobaculum* genus displayed strong positive correlations, whereas the *Bryobacter* and *Gemmatimonas* genera displayed strong negative correlations with metabolisms of amino acids and bile acids in GDM rats. The *Lactobacillus* and *Bifidobacterium* genera were positively correlated with gut metabolites. Overall, our results showed that metabolism signaling pathways of amino acids and bile acids are associated with the development of GDM. Probiotic supplements alleviate the pathology of GDM through the metabolism pathways of amino acids, bile acids, porphyrin, and chlorophyll.

## Introduction

Gestational diabetes mellitus (GDM) is the most common type of obstetric complication (Veeraswamy et al., 2012) and affects 14.8% of pregnant women in China (Gao et al., 2019). The incidence of GDM has increased globally due to poor dietary structure, unhealthy lifestyle changes, and environmental pollution (Ovesen et al., 2018; Rasmussen et al., 2020). GDM is not only associated with the adverse maternal-infant outcomes in the short term (Kurtzhals et al., 2018) but is also related to metabolic diseases in the woman and offspring in the long term, such as childhood obesity and type 2 diabetes (Kurtzhals et al., 2018; Ponnusamy et al., 2020). Therefore, effective measures to prevent and treat GDM are required.

The gut microbiome plays important roles in modulating host metabolism and immune cell development (He et al., 2020). Dysregulated gut microbiota is associated with diabetes, obesity, and other metabolic diseases (Qin et al., 2012). Metabolites also play important roles in revealing the gut microbiome and maintaining the health status of the host (Liu et al., 2017; Olson et al., 2018; Canfora et al., 2019; He et al., 2020). Abnormalities in gut microbiota and metabolomics in women are important for pathogenesis of GDM. GDM-related differential metabolites are mainly involved in metabolic pathways such as steroid hormone biosynthesis, metabolisms of amino acids, fatty acids, arachidonic acid, and butyric acid, and bile secretion (Li, 2019); however, gut metabolomics of GDM remain unclear.

Probiotics have positive effects on preventing and alleviating diabetes by regulating the intestinal microenvironment and immunity (Liu et al., 2017). Probiotic therapy may be a promising approach to improve glucose control and insulin resistance among pregnant women (de Brito Alves et al., 2019; Pan et al., 2021). Previously, our results showed that *Lactobacillus* and *Bifidobacterium* probiotic supplements could reduce the fasting blood glucose level of GDM rats by restoring the diversity of gut microbiota (Zheng et al., 2021). However, the effects of probiotic supplements on gut metabolomics in GDM are unclear. The associations between gut metabolites and microbiota in GDM rats are also unclear. Therefore, in this study, we analyzed gut metabolomic profiles of GDM rats and GDM rats treated with *Lactobacillus* and *Bifidobacterium* probiotic supplements. Associations between gut metabolites and microbiota in GDM rats were also studied.

## Materials and Methods

### Gestational Diabetes Mellitus Rats Mode and Probiotics Supplement

The procedures of GDM model construction and probiotics supplement can be accessed from our previous study (Zheng et al., 2021). Briefly, after adaptive feeding, 48-week-old female Sprague–Dawley rats were randomly divided into normal pregnant group that fed with ordinary feed and GDM model group that received high-fat and high-sugar feed for 6 weeks. Then, two female rats were paired with one male rat overnight. Pregnant rats were validated by vaginal secretions and recorded as embryonic day 1. The pregnant rats were further injected with streptozocin (25 mg/kg) to induce the development of GDM. Fasting blood glucose values higher than 7.8 mmol/L at embryonic day 4 were considered indicative of GDM rats. The GDM rats were intragastrically administrated with or without probiotic supplements of 0.5 g (low-dose group) or 1 g (high-dose group) for 15 days. Each gram of probiotic supplement contained 5 × 10^7^ colony-forming units (CFU) of *Lactobacillus rhamnosus LGG* and 1 × 10^8^ CFU of *Bifidobacterium animalis subspecies lactis* Bb12 (Life-space^®^, Australia). Finally, 10 rats in the normal pregnant group, eight rats in the GDM model group, eight GDM rats in the low-dose probiotics group, and nine GDM rats in the high-dose probiotics group were further studied. All animal procedures were approved by the Fujian University of Traditional Chinese Medicine (certificate number: SYXK 2019-0007; ethics approval number: FJTCM IACUC 2020020).

### Enzyme-Linked Immunosorbent Assay of Serum Parameters

Abdominal aortic blood was collected on embryonic day 19. Before blood extraction, a dose of 5 ml/kg of 20% uratan solution was injected intraperitoneally for anesthesia. Abdominal aortic blood was placed at 25°C room temperature for 2 h, centrifuged at a low temperature to obtain the serum sample, and finally stored at −80°C. Concentrations of interleukin-6 (IL-6), tumor necrosis factor–α (TNF-α), nitric oxide (NO), insulin, and low-density lipoprotein cholesterol (LDL-C), high-density lipoprotein cholesterol (HDL-C), and lipid factors including total cholesterol (TC) and triglyceride (TG) were measured using enzyme-linked immunosorbent assay (ELISA) kits (Boster Biological Technology Co., Ltd., Wuhan, Hubei, China for IL-6; ProteinTech^®^ Group, Chicago, IL, United States, for insulin and TNF-α; and Nanjing Jiancheng Bioengineering Institute Co., Ltd., Nanjing, Jiangsu, China, for NO, LDL-C, HDL-C, TC, and TG). The value of homeostasis model assessment–estimated insulin resistance (HOMA-IR) was also calculated.

### Histopathological Analysis

The pancreatic and colon tissues of rats were quickly collected and placed on ice. The tissues were fixed, embedded in paraffin, and stained with hematoxylin and eosin for histopathological analysis.

### Analysis of 16S rRNA Sequencing Data

The procedures and analysis method for 16S rRNA sequencing have been described in a previous study (Zheng et al., 2021). Raw sequence data are available in the Sequence Read Archive under the BioProject accession number PRJNA770477. The 16S rRNA sequencing was performed using Illumina HiSeq platform (Illumina, California, United States). Raw data were cleared using FLASH, TrimMomatic, and UCHIME software. In addition, operational taxonomic units (OTUs) of 16S rRNA sequencing data were clustered using the UCLUST software based on 97% similarity. The obtained OTUs were then matched with the SILVA database for taxonomic assignment.

### Fecal Samples Processing

At embryonic day 19, fecal samples were collected from each rat and stored in a −80°C refrigerator until metabolomic analysis. For fecal sample pretreatment, 50 mg of sample was weighed into an Eppendorf tube, and 1,000 μl of extract solution (acetonitrile:methanol:water = 2:2:1, with an isotopically labeled internal standard mixture) was added. After subjecting to a 30-s vortex, the samples were homogenized at 35 Hz for 4 min and sonicated for 5 min in an ice-water bath. The homogenization and sonication cycles were repeated three times. The samples were then incubated for 1 h at −40°C and centrifuged at 12,000 rpm for 15 min at 4°C. The resulting supernatant was transferred to a fresh glass vial for further analysis. The quality control sample was prepared by mixing an equal aliquot of the supernatant from all samples.

### Liquid Chromatography With Tandem Mass Spectrometry Analysis

LC-MS/MS analyses were performed using an ultrahigh-performance liquid chromatography system (Vanquish, Thermo Fisher Scientific) with a UPLC BEH amide column (2.1 × 100 mm, 1.7 μm) coupled to a Q Exactive HF-X mass spectrometer (Orbitrap MS, Thermo Fisher Scientific). The mobile phase consisted of 25 mmol/L of ammonium acetate and 25 mmol/L of ammonium hydroxide in water (pH = 9.75) and acetonitrile. The analysis was carried out with elution gradients as follows: 0–0.5 min, 95% B; 0.5–7.0 min, 95–65% B; 7.0–8.0 min, 65–40% B; 8.0–9.0 min, 40% B; 9.0–9.1 min, 40–95% B; and 9.1–12.0 min, 95% B. The column temperature was maintained at 30°C. The auto-sampler temperature was 4°C, and the injection volume was 3 μl.

The Q Exactive HF-X mass spectrometer was used for its ability to acquire MS/MS spectra in the information-dependent acquisition mode in the control of the acquisition software (Xcalibur, Thermo Fisher Scientific). In this mode, the acquisition software continuously evaluates the full-scan MS spectrum. Electrospray ionization source conditions were set as follows: sheath gas flow rate of 50 arbitrary units, aux gas flow rate of 10 arbitrary units, capillary temperature at 320^°^C, a full MS resolution of 60,000, MS/MS resolution of 7,500, collision energy of 10/30/60 in the normalized collision energy mode, and spray voltage of 3.5 kV (positive) or −3.2 kV (negative). Meanwhile, raw data were converted to the mzXML format using the ProteoWizard application and processed with an in-house program, which was developed using R and based on the XCMS platform, for peak detection, extraction, alignment, and integration. Then, an in-house MS2 database (BiotreeDB) was used for metabolite annotation. The cutoff for annotation was set at 0.3.

### Hierarchical Clustering Analysis

The hierarchical clustering analysis of the gut metabolites was performed using the pheatmap function in R software.

### Orthogonal Partial Least-Squares Discrimination Analysis

The Orthogonal Partial Least-Squares Discrimination Analysis (OPLS-DA) was used to analyze the metabolic patterns. The OPLS-DA permutation test was used to test the effect of the model fit. The *R*^2^Y (cum) index of the OPLS-DA permutation test was used to determine the accuracy of the model fit. The *Q*^2^ (cum) index refers to the predictive power of the model, and it is an evaluation parameter that is model-validated to prevent random fitting or overfitting. When the *R*^2^Y (cum) and *Q*^2^ (cum) indexes are closer to 1, the model fits well. In addition, if they are greater than 0.5, then the model is acceptable. On the basis of the threshold criteria of the projection of the first principal component of OPLS-DA > 1, an absolute fold change of < 0.5 or > 2 and a *P*-value of < 0.05 indicated a significant difference.

### Metabolic Signaling Pathways Analysis

Metabolic signaling pathways of gut metabolites were obtained from the Kyoto Encyclopedia of Genes and Genomes (KEGG) pathway database.^1^ The signaling pathways included more than four metabolites.

### Correlation Analysis and Co-occurrence Network of Target Metabolites and Gut Microbiota

The correlations of metabolites and the relative abundance of gut microbiota were analyzed using the Spearman’s correlation test through the psych package in the R software. The co-occurrence network of metabolites and gut microbiota was constructed using the Cytoscape software (version 3.1.1).

### Data Analysis

Statistical analysis and plotting of figures were conducted using GraphPad Prism (version 7.0), SPSS (version 25.0), and R programming language software (version 4.4.1). A normality test was performed to determine whether the data obeyed a normal distribution. If data obeyed a normal distribution, then a *t*-test or *post-hoc* test was performed to analyze the data. Otherwise, a non-parametric test was conducted. Statistical significance was set at *P* < 0.05, and data were presented as mean ± standard deviation (mean ± *SD*).

## Results

### Probiotic Supplements Restored Normal Serum Parameters, and Pancreatic and Colon Histology Are Influenced by Gestational Diabetes Mellitus

**FIGURE 1:**
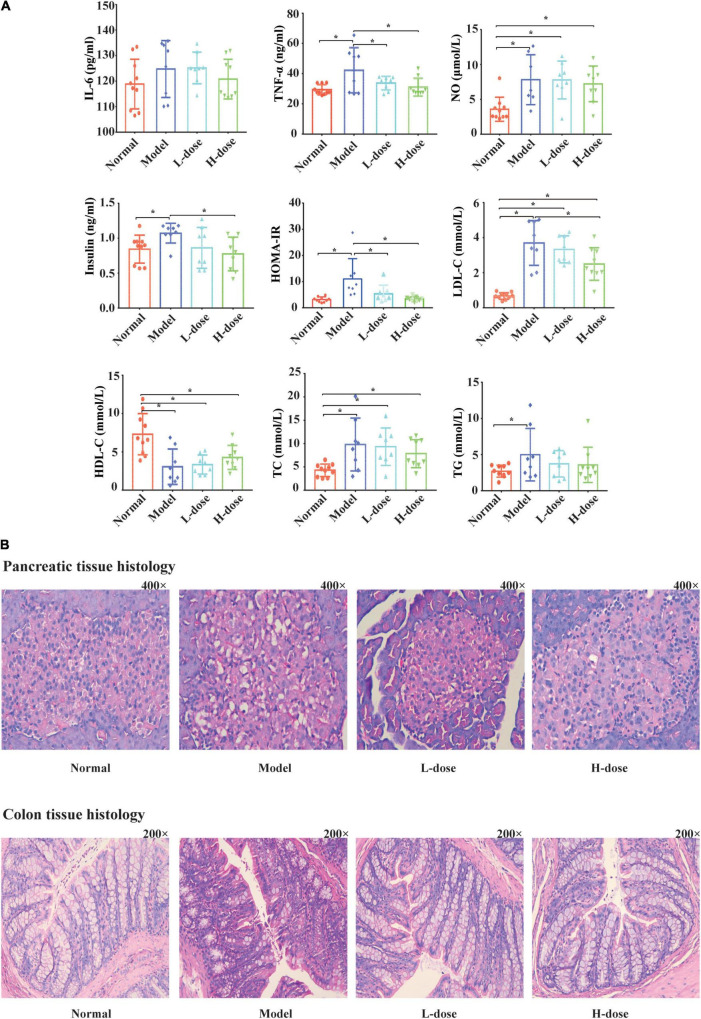
Probiotics supplements restore the normal serum parameters and pancreatic and colon histology influenced by GDM. **(A)** The concentrations of IL-6, TNF-α, NO, insulin, HOMA-IR, LDL-C, HDL, TC, and TG in GDM rats and GDM rats treated with *Lactobacillus* and *Bifidobacterium* probiotics supplements were tested. **(B)** The hematoxylin-eosin staining sections of the pancreas tissue and the colon tissue in GDM rats and GDM rats treated with probiotics supplements. *P < 0.05.

Previously, we successfully constructed a GDM rat model and demonstrated that *Lactobacillus* and *Bifidobacterium* probiotic supplements can reduce the fasting blood glucose level of GDM rats by restoring the diversity of gut microbiota (Zheng et al., 2021). Furthermore, using the same GDM model, we tested the effects of *Lactobacillus* and *Bifidobacterium* probiotic supplements on the concentrations of IL-6, TNF-α, NO, insulin, LDL-C, HDL-C, TC, and TG and estimated the value of HOMA-IR in the serum of GDM rats. In total, 10 normal pregnant rats, eight GDM rats, eight GDM rats with low-dosage probiotic supplements, and nine GDM rats with high-dosage probiotic supplements were tested. Compared to the normal pregnant rats, the concentrations of TNF-α, NO, insulin, LDL-C, TC, and TG (*P* = 0.002, *P* = 0.003, *P* = 0.041, *P* = 0.000, *P* = 0.000, and *P* = 0.000, respectively) and the value of HOMA-IR were increased in GDM rats (*P* = 0.000). Moreover, high-dose *Lactobacillus* and *Bifidobacterium* probiotic supplements significantly alleviated the concentrations of TNF-α, insulin, and LDL-C (*P* = 0.009, *P* = 0.011, and *P* = 0.007, respectively) and the value of HOMA-IR in GDM rats (*P* = 0.000). However, high-dose *Lactobacillus* and *Bifidobacterium* probiotic supplements did not alleviate the concentrations of NO, TC, and TG in GDM rats (*P* = 0.653, *P* = 0.046, and *P* = 0.002, respectively) (Figure 1A). In contrast, the concentrations of HDL-C were decreased in GDM rats (*P* = 0.000) and could not be further increased by the *Lactobacillus* and *Bifidobacterium* probiotic supplements (*P* = 0.794 and *P* = 0.358, respectively) (Figure 1A). Furthermore, the concentration of IL-6 was not significantly different among normal pregnant rats, GDM rats, and GDM rats with probiotic supplements (*P* < 0.05) (Figure 1A).

*Lactobacillus* and *Bifidobacterium* probiotic supplements could also influence the affected morphological and structural characteristics of pancreatic tissues in GDM rats. Compared to normal pregnant rats, the pancreatic islands of GDM rats were irregular and cyto-reduced and exhibited vacuolar degeneration. In addition, the capillary vessels of the pancreatic islands had undergone congestive expansion. After probiotic supplementation, the pancreatic islands of the rats were clearer, had less vacuolar degeneration, and showed cellular increase compared to these parameters in GDM rats (Figure 1B).

The morphological and structural characteristics of the colon tissues of GDM rats were also analyzed (Figure 1B). The colon mucous membrane and cell–cell junction of the epithelial cells of GDM rats were significantly damaged. The cryptostructures of colon tissues partially disappeared, and the beaker cells were reduced. After probiotic supplementation, the colon mucous membrane of rats was more complete and displayed queuing discipline compared to that in the GDM model group, and the numbers of cryptostructures and beaker cells were further increased.

### Altered Metabolites in Gestational Diabetes Mellitus Rats

One possible mechanism of *Lactobacillus* and *Bifidobacterium* probiotic supplements restoring the normal serum parameters and pancreatic and colon histology influenced by GDM is the regulation of gut metabolites. Fecal metabolomic studies facilitate the interpretations of the metabolic interactions between the host, diet, and gut microbiota and provide functional data on the microbiome. Therefore, in this study, we further explored the influence of probiotic supplements on gut metabolites in the fecal samples of GDM rats using LC-MS/MS.

**FIGURE 2:**
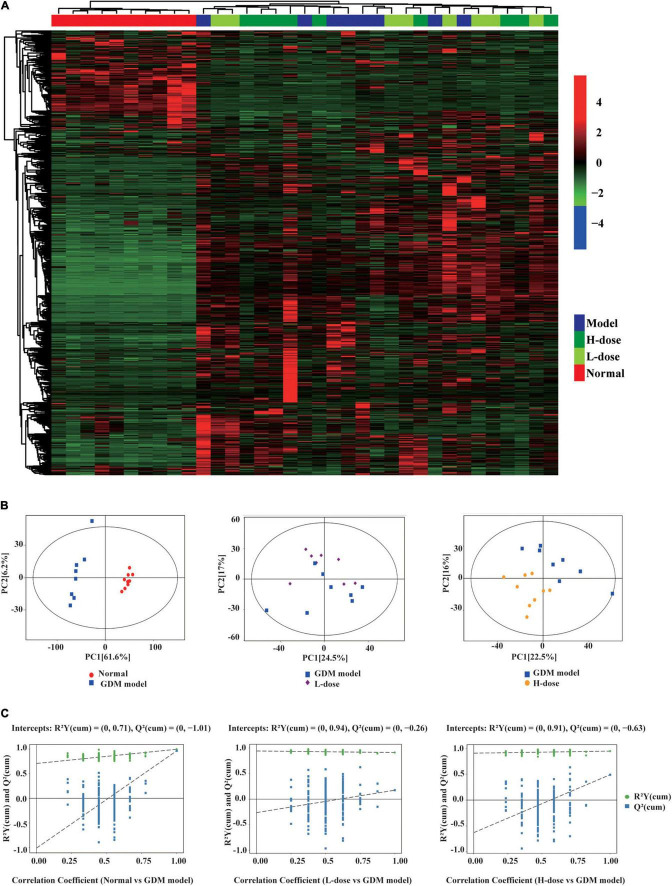
The gut metabolites feature of GDM rats and GDM rats treated with probiotics. **(A)** Hierarchical clustering analysis of 999 metabolites in GDM rats and GDM rats treated with *Lactobacillus* and *Bifidobacterium* probiotics supplements. The OPLS-DA analysis **(B)** and the OPLS-DA permutation test **(C)** of the gut metabolites in GDM rats and GDM rats treated with probiotics supplements.

In total, 999 gut metabolites were detected. Normal rats were clearly distinguished from the GDM rats based on the levels of 999 gut metabolites by using the unsupervised clustering heatmap (Figure 2A). In addition, in the OPLS-DA score plots, normal pregnant rats and GDM rats were clearly separated from each other (Figure 2B). Similarly, the OPLS-DA permutation test indicated that the classifications of normal and GDM rats were robust and exhibited good fitness and prediction [(*R*^2^Y (cum) = (0, 0.71), Q2 (cum) = (0, −1.01)] (Figure 2C). Moreover, compared to normal rats, the levels of most gut metabolites were increased in GDM rats (Figure 2A).

However, in the unsupervised clustering heatmap, the GDM rats were indistinguishable from the GDM rats treated with low- or high-dose *Lactobacillus* and *Bifidobacterium* probiotic supplements (Figure 2A). In addition, in the OPLS-DA score plots and in the OPLS-DA permutation test, GDM rats and GDM rats with low probiotic supplements could not be distinctly separated from each other (Figures 2B,C). In contrast, GDM rats with high probiotic supplements represented a separate group in the OPLS-DA score plots and in the OPLS-DA permutation test (Figures 2B,C).

### Altered Metabolites in Gestational Diabetes Mellitus Rats Treated With Probiotics

**FIGURE 3:**
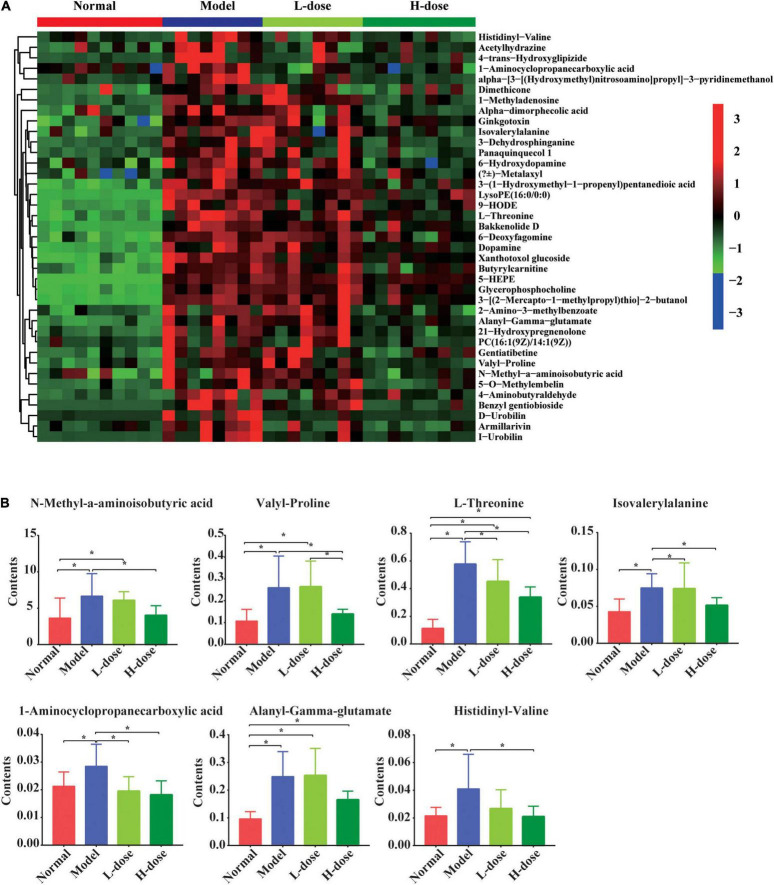
The influenced gut metabolites after *Lactobacillus* and *Bifidobacterium* probiotics supplements in GDM rats. **(A)** Hierarchical clustering analysis of 44 metabolites in GDM rats and GDM rats treated with *Lactobacillus* and *Bifidobacterium* probiotics supplements. **(B)** The comparison of seven amino acids by the ANOVA/Kruskal–Wallis and *post-hoc* test. *P < 0.05.

Although most of the altered metabolites in GDM rats could not be restored by *Lactobacillus* and *Bifidobacterium* probiotic supplements, partial metabolites were significantly influenced by probiotic supplements. We detected 44 metabolites that were increased in GDM rats, and their levels were alleviated by high-dose *Lactobacillus* and *Bifidobacterium* probiotic supplements (Figure 3A).

The levels of N-methyl-α-aminoisobutyric acid, L-proline, L-threonine, isovalerylalanine, 1-aminocyclopropanecarboxylic acid, alanyl-gamma-glutamate, and histidinyl-valine were shown in the box plots. The concentrations of N-methyl-α-aminoisobutyric acid, valyl-proline, L-threonine, isovalerylalanine, 1-aminocyclopropanecarboxylic acid, alanyl-gamma-glutamate, and histidinyl-valine concentrations were increased in GDM rats (*P* = 0.008, *P* = 0.002, *P* = 0.000, *P* = 0.004, *P* = 0.015, *P* = 0.000, and *P* = 0.008, respectively). Moreover, high-dose *Lactobacillus* and *Bifidobacterium* probiotic supplements significantly reduced the concentrations of these metabolites in GDM rats (*P* < 0.05) (Figure 3B), suggesting that probiotic supplements restored normal serum parameters and pancreatic and colon histology influenced by GDM through the regulation of these metabolites.

### Changes in Metabolism Signaling Pathways of Amino Acids and Bile Acids in Gestational Diabetes Mellitus Rats

The changes in metabolites in the GDM rats were further studied. There were 499 metabolites that significantly changed in GDM rats compared to normal pregnant rats. Furthermore, the top 20 most significantly changed metabolites in GDM rats vs. normal pregnant rats were shown in Table 1. Those significantly changed metabolites in GDM rats was dihydroisoalantolactone, followed by (E)-2-Butyl-2-octenal and 4-Hydroxy-4-(3-pyridyl)-butanoic acid.

**TABLE 1:** The top 20 most significantly changed metabolites in normal pregnant rats vs. GDM rats.

MS2 name	RT	MZ	VIP	*P*-value	Fold change
Dihydroisoalantolactone	164.29	235.17	1.55	3.33017E-05	13574.95
(E)-2-Butyl-2-octenal	26.00	183.17	1.55	3.48987E-06	1914.06
4-Hydroxy-4-(3-pyridyl)-butanoic acid	276.66	182.08	1.55	0.000113978	10738.12
Ginsenoyne A linoleate	291.61	521.39	1.55	0.000731182	458.18
Tetraphyllin B	385.17	288.11	1.54	4.58102E-06	42.55
L-Palmitoylcarnitine	191.30	400.34	1.54	1.30394E-05	82.88
18-Nor-4(19),8,11,13-abietatetraene	236.83	255.21	1.54	0.00293506	2975.01
Vaccenyl carnitine	188.27	426.36	1.54	8.58595E-06	80.69
3,4-Dihydroxy-2-hydroxymethyl-1-pyrrolidinepropanamide	422.04	205.12	1.54	2.43937E-06	18.21
(3R,6′Z)-3,4-Dihydro-8-hydroxy-3-(6-pentadecenyl)-1H-2-benzopyran-1-one	213.94	373.27	1.54	0.000121065	47.22
6,7-Dihydro-5-methyl-5H-cyclopenta[b]pyrazine	252.91	135.09	1.51	6.03717E-07	0.18
[10]-Gingerdione	52.02	349.24	1.51	0.000162074	0.03
5-Aminopentanal	257.63	102.09	1.48	5.90244E-07	0.22
Tryptophyl-Valine	307.05	304.16	1.47	5.79417E-07	0.24
Linatine	383.38	260.12	1.47	6.24363E-07	0.24
1,9-Nonanedithiol	289.66	193.11	1.47	2.31553E-05	0.22
3-Methylpyrrolo[1,2-a]pyrazine	144.88	134.07	1.47	3.88429E-06	0.19
2-Methoxycanthin-6-one	307.70	251.08	1.47	4.322E-06	0.27
Calystegin A3	77.68	160.10	1.47	4.32426E-13	0.09
Acetylcholine	262.24	146.12	1.47	1.21646E-07	0.16

*RT, retention time; MZ, mass-to-charge ratio; VIP, PLS-DA first principal component variable importance in projection; P-value, t-test significance; the Fold change was < 0.5 or > 2.*

**FIGURE 4:**
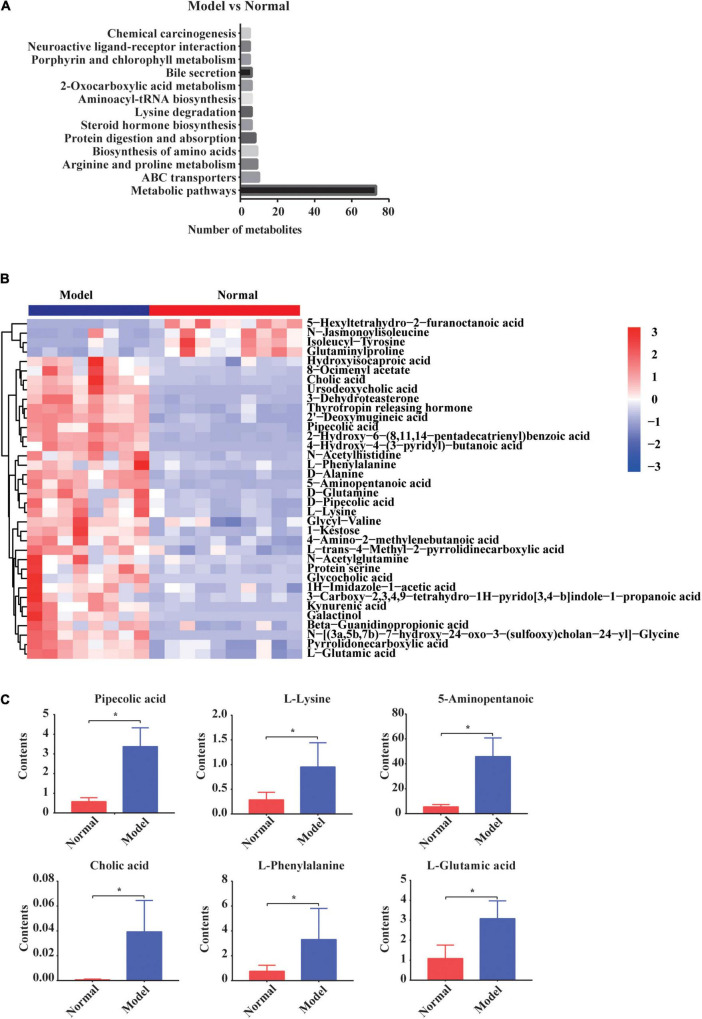
The changed gut metabolites and signaling pathways in normal pregnant rats vs. GDM rats. **(A)** Gut metabolites’ signaling pathways in KEGG Database. **(B)** Hierarchical clustering analysis of 36 metabolites in GDM rats. **(C)** The comparison of amino acids and bile acids that participated in gut metabolites’ signaling pathways by independent samples *t*-test or non-parametric test. *P < 0.05.

Next, the KEGG signaling pathways associated with the significantly altered metabolites in GDM rats vs. normal pregnant rats were studied. Although these metabolites were diverse, they were mainly concentrated in signaling pathways of amino acids, organic acids, and bile acids (Figure 4A). Metabolites including amino acids, organic acids, and bile acids and glucuronide metabolites were further depicted in the heatmap (Figure 4B). The concentrations of most of the metabolites were increased in GDM rats. For example, pipecolic acid, l-lysine, 5-aminopentanoic acid, L-phenylalanine, L-glutamic acid, and cholic acid from porphyrin and chlorophyll metabolism, biosynthesis of amino acids, and bile secretion signaling pathways were all significantly increased in GDM rats compared to their levels in normal pregnant rats (*P* = 0.000, *P* = 0.006, *P* = 0.006, *P* = 0.000, *P* = 0.003, and *P* = 0.015, respectively) (Figure 4C).

### Metabolism Pathways of Porphyrin and Chlorophyll Were Associated With the Altered Metabolites Following Probiotic Supplementation

The changes in metabolites in GDM rats treated with *Lactobacillus* and *Bifidobacterium* probiotics were further studied. Compared to the metabolites in the GDM rats, low-dosage probiotic supplements induced the changes of 56 metabolites and high-dosage probiotic supplements induced the changes of 102 metabolites. Moreover, the top 20 most significantly changed metabolites in the GDM rats vs. GDM rats with low-dosage probiotic supplementation and GDM rats vs. GDM rats with high-dose probiotic supplementation are shown in Tables 2, 3, respectively. LysoPE and 2-aminoacrylic acid were significantly changed after low-dose probiotic supplementation, whereas [4]-gingerdiol 3,5-diacetate and propionylcarnitine were significantly changed after high-dose probiotic supplementation.

**TABLE 2:** The top 20 most significantly changed metabolites in GDM rats treated with lose-dose probiotics supplements vs. GDM rats.

MS2 name	RT	MZ	VIP	*P*-value	Fold change
LysoPE (0:0/20:5(5Z,8Z,11Z,14Z,17Z))	108.85	500.27	2.45	0.030380947	10.67
2-Aminoacrylic acid	408.26	88.04	2.18	0.020937481	2.91
2,5-Dimethyloxazole	150.27	98.06	2.15	0.02726155	2.82
Kudzusaponin SA2	307.74	945.50	2.04	0.047526679	3.72
Polysorbate 60	257.00	435.29	1.73	0.030919879	2.31
Clupanodonyl carnitine	235.12	474.36	1.72	0.015841942	3.34
Saxitoxin	288.47	300.14	1.60	0.031002897	3.44
PI (16:1(9Z)/18:0)	209.56	837.56	1.37	0.046339361	2.62
N-Hydroxy-L-tyrosine	289.75	198.08	2.44	0.040165452	0.36
(R)-Salsolinol	190.40	180.10	2.43	0.021349123	0.26
L-Urobilin	289.95	595.35	2.32	0.043622447	0.08
Quinoline-4,8-diol	186.32	162.05	2.21	0.041281558	0.38
Octadecanedioic acid	151.91	315.25	2.12	0.032593502	0.12
D-Urobilin	255.62	589.30	2.12	0.038878374	0.12
Dukunolide C	402.78	541.17	2.10	0.015048225	0.22
alpha-[3-[(Hydroxymethyl) nitrosoamino]propyl]-3-pyridinemethanol	296.84	226.12	1.94	0.039346005	0.33
2,3-Dimethyl-5-(2-propenyl) pyrazine	230.60	149.11	1.65	0.04508631	0.48

*RT, retention time; MZ, mass-to-charge ratio; VIP, PLS-DA first principal component variable importance in projection; P-value, t-test significance; the Fold change was < 0.5 or > 2.*

**TABLE 3:** The top 20 most significantly changed metabolites in GDM rats treated with high-dose probiotics supplements vs. GDM rats.

MS2 name	RT	MZ	VIP	*P*-value	Fold change
-Gingerdiol 3,5-diacetate	255.47	370.22	2.30	0.000782449	3.16
Propionylcarnitine	291.02	218.14	2.29	0.005544317	2.81
LysoPE [0:0/20:5(5Z,8Z,11Z,14Z,17Z)]	108.85	500.27	2.27	0.016205496	4.90
2-Aminoacrylic acid	408.26	88.04	2.24	0.03169583	2.93
Acetamidopropanal	411.68	116.07	2.23	0.009543001	2.22
Carpaine	284.96	479.38	2.20	0.045061999	14.98
Lysyl-Valine	533.40	246.18	2.13	0.045907893	5.69
2,9-Bis (3-methyl-2E-pentenoyl)-2b,9a-dihydroxy-4Z,10(14)-oplopadien-3-one	131.70	443.27	2.13	0.04138836	8.27
Lysyl-Leucine	533.48	260.20	2.06	0.019666532	3.68
Saxitoxin	288.47	300.14	2.04	0.000426356	4.06
L-Leucine	344.89	132.10	2.62	0.00245802	0.21
Aminoadipic acid	82.73	162.08	2.49	0.003544451	0.47
3-Dehydrosphinganine	107.48	300.29	2.46	0.002750615	0.28
L-alpha-Amino-1H-pyrrole-1-hexanoic acid	361.76	197.13	2.42	0.00013354	0.45
Xanthotoxol glucoside	320.46	365.08	2.42	0.000384492	0.59
I-Urobilin	298.02	591.32	2.41	0.018958387	0.14
5-(2-Furanyl)-3,4-dihydro-2H-pyrrole	223.18	136.08	2.40	0.000761589	0.64
(R)-Salsolinol	190.40	180.10	2.40	0.010778167	0.14
Armillarivin	323.14	385.20	2.40	0.005431808	0.20
3-Formyl-6-hydroxyindole	69.03	162.05	2.36	0.039772211	0.13

*RT, retention time; MZ, mass-to-charge ratio; VIP, PLS-DA first principal component variable importance in projection; P-value, t-test significance; the Fold change was < 0.5 or > 2.*

**FIGURE 5:**
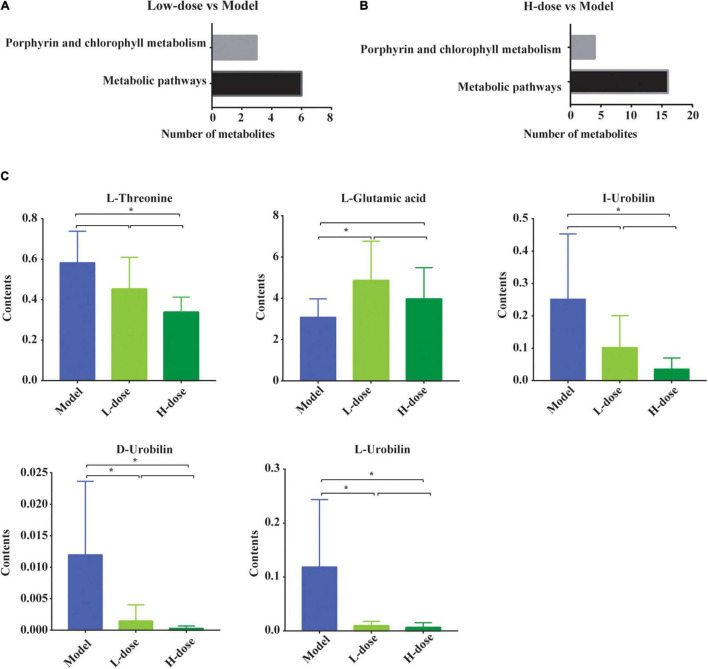
The changed gut metabolites and pathways in GDM rats treated with probiotics. **(A,B)** The porphyrin and chlorophyll metabolism pathway and metabolic pathway in KEGG Database. **(C)** The comparison of gut metabolites which participated in porphyrin and chlorophyll metabolism pathway and metabolic pathway by the *post-hoc* test or non-parametric test. *P < 0.05.

Next, the KEGG signaling pathways associated with the significantly changed metabolites in GDM rats vs. GDM rats with low-dose probiotic supplementation and GDM rats vs. GDM rats with high-dose probiotic supplementation were analyzed. Compared to GDM rats, the metabolites induced by low- or high-dose probiotic supplementation were concentrated on the metabolism pathways of porphyrin and chlorophyll (Figures 5A,B). Interestingly, the metabolism pathways of porphyrin and chlorophyll were also associated with the altered metabolites in GDM rats vs. normal pregnant rats (Figure 4A). We further showed that the levels of the metabolites L-threonine, I-urobilin, D-urobilin, and L-urobilin from the metabolism pathways of porphyrin and chlorophyll were decreased by high-dose probiotic supplementation in GDM rats (*P* = 0.001, *P* = 0.002, *P* = 0.002, and *P* = 0.005, respectively) (Figure 5C). In addition, D-urobilin and L-urobilin levels were also decreased by low-dose probiotic supplementation in GDM rats (*P* = 0.005 and *P* = 0.006, respectively) (Figure 5C), whereas L-glutamic acid level was decreased by low-dosage probiotic administration in GDM rats (*P* = 0.026) (Figure 5C).

### Changed Metabolites From Metabolism Signaling Pathways of Amino Acids and Bile Acids After Probiotic Supplementation

**FIGURE 6:**
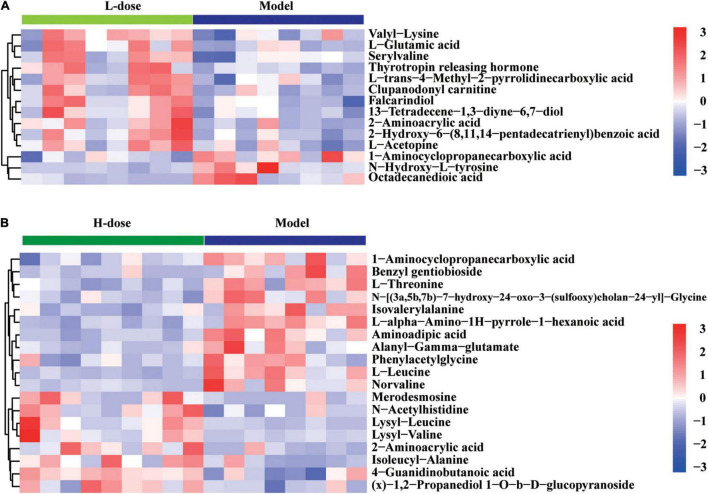
The changed amino acids and bile acids in GDM rats treated with probiotics supplements. **(A)** Hierarchical clustering analysis of 14 amino acids and bile acids in GDM rats treated with low-dose probiotics supplements vs. GDM rats. **(B)** Hierarchical clustering analysis of 19 amino acids and bile acids in GDM rats treated with high-dose probiotics supplements vs. GDM rats.

Although the significantly altered metabolites in GDM rats vs. GDM rats with *Lactobacillus* and *Bifidobacterium* probiotic supplementation were not enriched with metabolism signaling pathways of amino acids and bile acids, some metabolites from these pathways were still influenced by probiotic supplements. We showed that the concentration of 2-aminoacrylic acid was increased in GDM rats following supplementation with low or high dosages *of Lactobacillus* and *Bifidobacterium* probiotics, whereas that of 1-aminocyclopropanecarboxylic acid was decreased in GDM rats following supplementation with low or high dosages of probiotics (Figures 6A,B). Moreover, the concentrations of valyllysine, L-glutamic acid, serylvaline, thyrotropin-releasing hormone, clupanodonyl carnitine, and falcarindiol were significantly increased after the administration of low-dose *Lactobacillus* and *Bifidobacterium* probiotic supplements (Figure 6A). In contrast, the concentrations of 1-aminocyclopropanecarboxylic acid, benzyl-gentiobioside, L-threonine, isovalerylalanine, aminoadipic acid, alanyl-gamma-glutamate, phenylacetylglycine, L-leucine, and norvaline were significantly reduced by high-dose probiotic supplements (Figure 6B).

### Correlations of Gestational Diabetes Mellitus-Related Gut Microbiota and Metabolites

**FIGURE 7:**
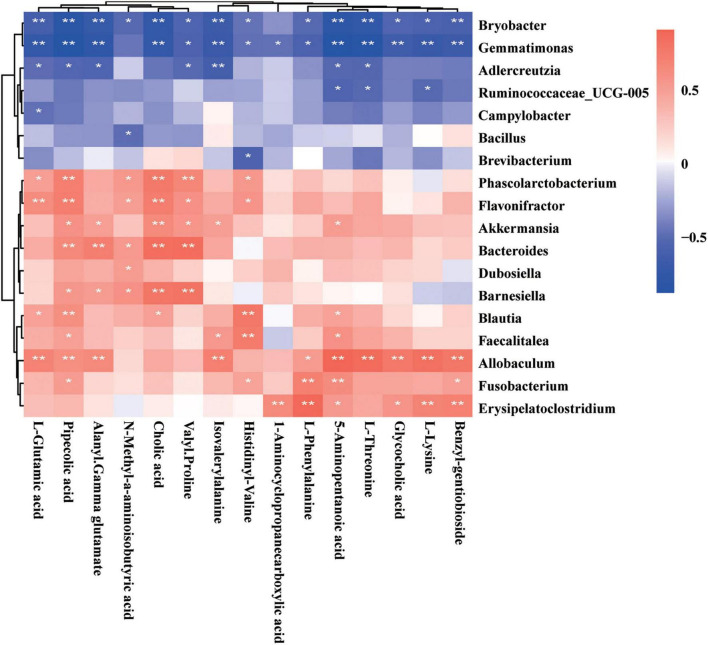
Spearman’s correlation analysis of metabolites and gut microbiota. The *r*-values were denoted with graduated colors, and red and blue grids indicated positive and negative correlations, respectively (*P < 0.05 and ** *P* < 0.01).

Fecal metabolomics may reflect the functions of the gut microbiome. The altered metabolites in GDM rats may be perturbed by the gut microbiome during GDM pathogenesis. Previously, we studied gut microbiota in GDM rats and GDM rats with *Lactobacillus* and *Bifidobacterium* probiotic supplements (Zheng et al., 2021). Next, we explored the functional correlations between gut microbiota perturbations and metabolite changes. We observed strong correlations between the gut bacterial families and metabolites assigned to the metabolism of amino acids and bile acids in GDM rats, as determined by the Spearman’s correlation coefficient. The gut bacterial OTUs belonging to the genus *Allobaculum* displayed strong positive correlations (*P* < 0.05), whereas the genera *Bryobacter* and *Gemmatimonas* displayed strong negative correlations with the metabolism of amino acids and bile acids, such as L-glutamic acid, pipecolic acid, alanyl-gamma glutamate, isovalerylalanine, L-phenylalanine, 5-aminopentanoic acid, L-threonine, glycocholic acid, and lysine (*P* < 0.05) (Figure 7). Moreover, the metabolites pipecolic acid, 5-aminopentanoic acid, and cholic acid were associated with most of the gut bacterial genera in GDM rats (*P* < 0.05) (Supplementary Figure 1A).

To further explore the reciprocal synergistic relationships between gut microbiota and metabolites, we constructed a co-occurrence network based on the correlations of the gut bacterial genus and its metabolites in GDM rats (Figure 7). We found that metabolites 5-aminopentanoic acid, alanyl-gamma glutamate, benzyl-gentiobioside L-phenylalanine, and the gut bacterial genera *Phascolarctobacterium*, *Barnesiella*, and *Blautia* were at the hub of the co-occurrence network, whereas other gut bacterial genera and their metabolites were connected with those hub factors (Supplementary Figure 1A).

### *Lactobacillus* and *Bifidobacterium* Were Positively Correlated With the Gut Metabolites

**FIGURE 8:**
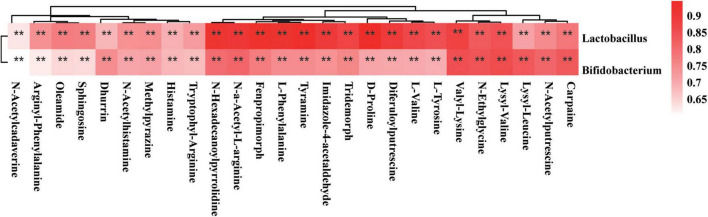
Spearman’s correlation analysis of *Lactobacillus* and *Bifidobacterium* genus and metabolites. The *r*-values were denoted with graduated colors, and red grids indicated positive correlations (*P < 0.05 and **P < 0.01).

Lactobacillus *rhamnosus* LGG and *Bifidobacterium lactis* Bb12 were used as probiotic supplements in this study. Therefore, we tested the correlations of the *Lactobacillus* and *Bifidobacterium* genera with gut metabolites. The Spearman’s correlation coefficient of the OTU of *Lactobacillus* and *Bifidobacterium* genera and concentrations of 999 detected gut metabolites were determined. On the basis of *r*-values of > 0.6 and *P*-values of < 0.01, the *Lactobacillus* genus was positively correlated with 90 gut metabolites and the *Bifidobacterium* genus was positively correlated with 71 gut metabolites. None of the gut metabolite showed a negative correlation with the OTUs of *Lactobacillus* or *Bifidobacterium* genera. The correlations of 26 gut metabolites with *Lactobacillus* and *Bifidobacterium* genera are shown in a heatmap (Figure 8); these include the amino acid metabolites arginyl-phenylalanine, L-phenylalanine, D-proline, lysyl-valine, valyl-lysine, lysyl-leucine, L-tyrosine, N-α-acetyl-L-arginine, and tryptophyl-arginine. The co-occurrence network also revealed the connections of *Lactobacillus* and *Bifidobacterium* probiotic supplements with amino acid metabolism (Supplementary Figure 1B). In addition, other metabolites, such as histamine, oleamide, fenpropimorph, dhurrin, and tyramine, were also correlated with the OTUs of the *Lactobacillus* or *Bifidobacterium* genera (Supplementary Figure 1B).

## Discussion

Probiotics have a mild, stable, and effective influence on controlling blood glucose (Peng et al., 2018; Zhang et al., 2019; Masulli et al., 2020). However, the effects of probiotics on gut metabolites in GDM rats are not clear (Yao et al., 2017). Our results showed that high-dose *Lactobacillus* and *Bifidobacterium* probiotic supplements can decrease the concentrations of TNF-α, insulin, and LDL-C and the value of HOMA-IR in GDM rats. After *Lactobacillus* and *Bifidobacterium* probiotic intervention, the morphological and structural characteristics of pancreatic and colon tissues were similar to those in the normal pregnant group. In addition, after probiotic supplementation, GDM model rats showed significant changes in gut microbiome composition and gut metabolic profiles. Moreover, the perturbed gut microbiota was strongly associated with changes in gut microbiota-related metabolites, suggesting that probiotic supplements can substantially alter the metabolomic profile of the gut microbiome and adjust host metabolite homeostasis.

Our results from a previous study (Zheng et al., 2021) and this study further verified that *L. rhamnosus* LGG and *B. lactis* Bb12 used as probiotic supplements can reduce blood glucose and insulin resistance in GDM rats and also improve the morphological and structural characteristics of their pancreatic and colon tissues. Potential mechanisms of probiotic supplements regulating blood glucose were mainly manifested in regulating the structure of gut microbiota and gut metabolites and then reducing the level of fasting blood glucose and insulin resistance. In addition, insulin resistance has been regarded as a cause of endothelial dysfunction in diabetes (Zhang et al., 2012).

Changes in gut metabolomics are associated with intestinal dysbacteriosis in the development of obesity and metabolic syndrome (Qin et al., 2012; Karlsson et al., 2013). Moreover, metabolic alterations can further aid in the exploration of the pathological mechanisms of GDM. We found that the fecal metabolic profile of GDM rats was significantly different from that of normal pregnant rats. In total, 999 gut metabolites were detected in the feces. Forty-four metabolites in GDM rats had increased concentrations, which were reduced by probiotic supplements, including amino acids, bile acids, and glucuronide. Changes in metabolites in GDM rats were associated with the metabolism signaling pathways of amino acids and bile acids. Metabolites from metabolism signaling pathways of amino acids and bile acids were influenced by probiotic supplements. Changes in metabolites after probiotic supplementation were associated with the metabolism pathways of porphyrin and chlorophyll.

The fecal metabolic profiles of GDM rats were significantly different from those of normal pregnant rats in the metabolism pathways of amino acids and bile acids. The identified amino acids participating in the development of GDM rats included 1-pyrroline, creatinine, cytosine, 2-hydroxypyridine, choline, 2,6-dimethylpyridine, and quinoline. These results are similar to those of previous studies (Wang et al., 2020; Zhao et al., 2020; Lu et al., 2021) that suggest that fecal gut metabolomics of women with GDM was distinct from that of healthy pregnant women, and the levels of amino acids were disrupted in the former. Moreover, the levels of amino acids in early pregnancy are potential biomarkers for predicting subsequent GDM (Jiang et al., 2020). Consistent with previous reports (Lu et al., 2021), we found that, compared to the metabolic profiles of normal pregnant rats, the bile secretion signaling pathways were significantly increased in GDM rats. Both our results and previous studies suggest that bile acids participate in the development of GDM. Serum bile acids in early pregnancy like deoxycholic acid ≤ 0.28 nmol/ml or glycoursodeoxycholic acid ≤ 0.07 nmol/ml were significantly associated with increased risk of GDM (Li et al., 2018). Bile acids play important roles in glucose and lipid metabolism (Vítek and Haluzik, 2016). Furthermore, the relationship between bile acid metabolism and the gut microbiome is mutual (Vítek and Haluzik, 2016). On the one hand, gut microbiota can metabolize bile acids. On the other hand, bile acids can affect the composition of gut microbiota (Vítek and Haluzik, 2016). Our results indicated the relationship between gut microbiota and bile acid and the potential influences of their interaction on host glucose and lipid metabolism in GDM.

A balanced structure of gut microbiota and its metabolites are beneficial to the health of the host; however, the poor interaction between the host and its gut microbiota can lead to metabolic dysfunction (Hills et al., 2019). Disturbances in gut microbiota might contribute to GDM pathogenesis by modulating the metabolism signaling pathways of amino acids and bile acids (Wang et al., 2020). For example, gut microbiota perturbations of the *Bacteroides* and *Bifidobacterium* genera displayed strong correlations with amino acid metabolism, and the *Bifidobacterium* genus was also positively associated with bile acid metabolism. *Allobaculum* genus displayed strong positive correlations, whereas the *Bryobacter* and *Gemmatimonas* genera displayed strong negative correlations with amino acid and bile acid metabolism in GDM rats. *Lactobacillus*, *Bifidobacterium*, and *Akkermansia* are widely used to aid in weight loss and ameliorate metabolic diseases such as obesity and diabetes (Park et al., 2019; Yan et al., 2019; Li et al., 2020). *L. rhamnosus* LGG and *B. lactis* Bb12 were used as probiotic supplements to treat GDM model rats in this study. Consistent with the findings from our previous study (Zheng et al., 2021) as well as the present study, a previous randomized controlled trial found that *L. rhamnosus* HN001 supplementation in early pregnancy decreased GDM prevalence and fasting conjugated bile acids, and these bile acids were positively correlated with fasting glucose and fasting insulin, suggesting that they play important roles in improving the glucose metabolism in pregnant women (Chen et al., 2021).

In summary, 16S RNA sequencing and metabolomic analysis conducted in this study revealed the effect of probiotic supplements (*Lactobacillus* and *Bifidobacterium*) on the gut microbiota and gut microbiome of GDM rats and the metabolic status of normal pregnant rats and GDM rats. In addition, this work extended our insights into the relationship among gut microbiota, host metabolism, and GDM rats, pointing to possible future modalities to treat GDM by targeting gut microbiota or gut metabolites. More research is needed to further identify the bacterial species that play a key role in the metabolism of GDM. Overall, this study indicated that probiotic supplementation might be a novel approach to reduce blood glucose and insulin resistance and regulate gut microbiota or gut metabolites in GDM rats. Furthermore, this study provides insights into the applicability of probiotic supplementation in the treatment of GDM.

## Data Availability Statement

The raw data supporting the conclusions of this article will be made available by the authors, without undue reservation.

## Ethics Statement

All animal procedures were approved by the Fujian University of Traditional Chinese Medicine (Certificate number: SYXK 2019-0007; Ethics approval number: FJTCM IACUC 2020020).

## Author Contributions

Q-XZ and H-WW analyzed data, plotted figures, drafted, and revised the manuscript. Q-XZ and X-MJ designed this project and performed coordination. LG revised important scholarly content of our manuscript. Y-TL, X-YJ, P-PH, FC, and X-QC performed the rat experiments, collected, and analyzed samples. All authors contributed to the article and approved the submitted version.

## Conflict of Interest

The authors declare that the research was conducted in the absence of any commercial or financial relationships that could be construed as a potential conflict of interest.

## Publisher’s Note

All claims expressed in this article are solely those of the authors and do not necessarily represent those of their affiliated organizations, or those of the publisher, the editors and the reviewers. Any product that may be evaluated in this article, or claim that may be made by its manufacturer, is not guaranteed or endorsed by the publisher.

## Footnotes

1
https://www.kegg.jp/kegg/pathway.html

## Supplementary Material

Supplementary Figure 1 Reciprocal synergistic relationships of metabolites and gut microbiota in GDM rats. **(A)** The co-occurrence network of metabolites and gut microbiota. Yellow points represented gut metabolites, and green points represented gut microbiota. Red lines represented positive correlations, and blue lines represented negative correlations. **(B)** The co-occurrence network of *Lactobacillus* and *Bifidobacterium* genus and metabolites. Yellow points represented gut metabolites, and green points represented gut microbiota. Red lines represented positive correlations.
