# Supplementary figures and images for: Changes in the Gut Metabolic Profile of Gestational Diabetes Mellitus Rats Following Probiotic Supplementation
Source: Front Microbiol. 2022 Apr 8;13:779314. doi: 10.3389/fmicb.2022.779314 (PMC9024396; doi:10.3389/fmicb.2022.779314)

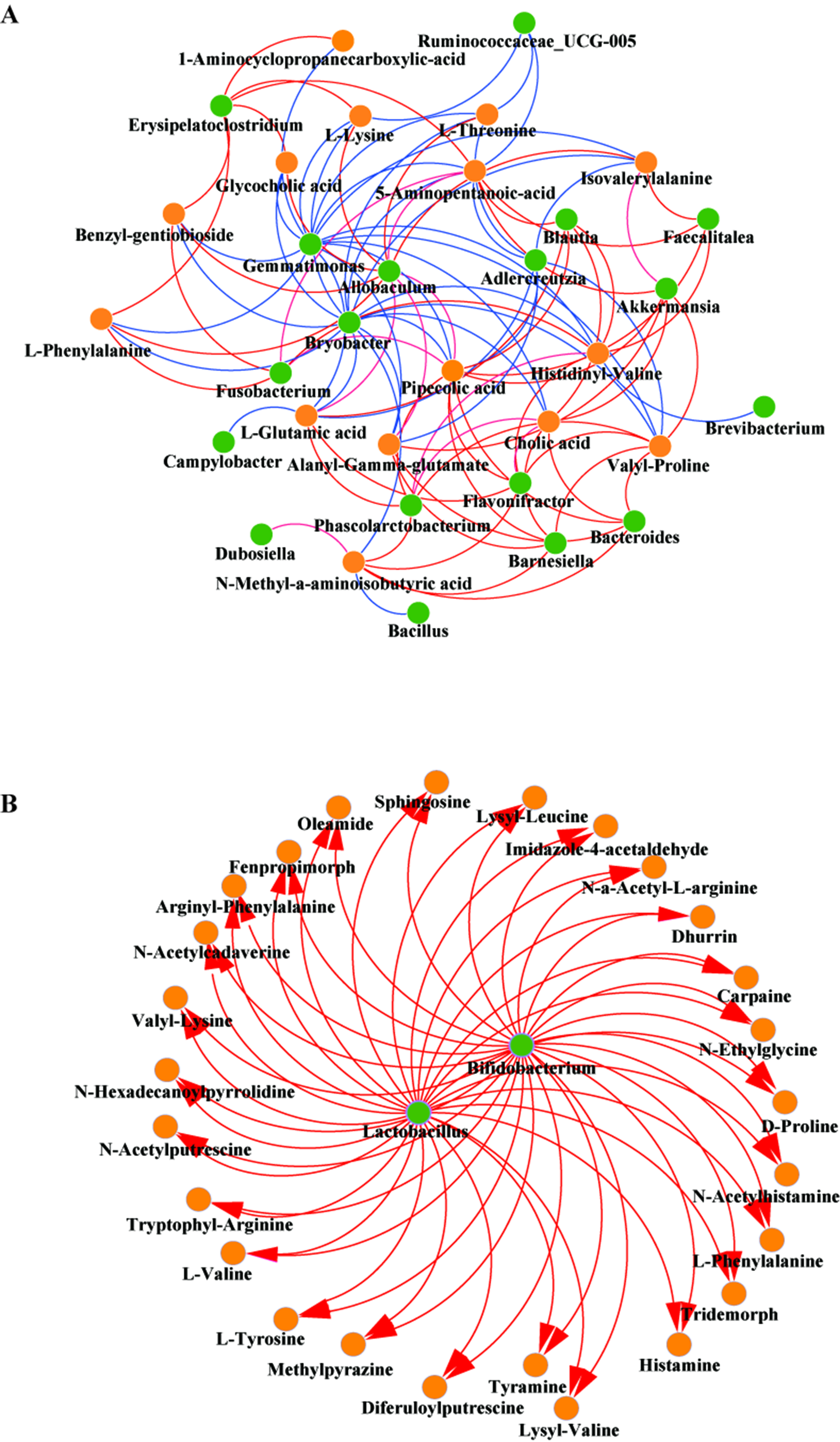

Supplement: Supplementary Figure 1 — Reciprocal synergistic relationships of metabolites and gut microbiota in GDM rats. (A) The co-occurrence network of metabolites and gut microbiota. Yellow points represented gut metabolites, and green points represented gut microbiota. Red lines represented positive correlations, and blue lines represented negative correlations. (B) The co-occurrence network of Lactobacillus and Bifidobacterium genus and metabolites. Yellow points represented gut metabolites, and green points represented gut microbiota. Red lines represented positive correlations. [file Image_1.tif]
